# Toxicological evidence integration to confirm the biological plausibility of the association between humidifier disinfectant exposure and respiratory diseases using the AEP-AOP framework

**DOI:** 10.4178/epih.e2024060

**Published:** 2024-07-07

**Authors:** Ha Ryong Kim, Jun Woo Kim, Jong-Hyeon Lee, Younghee Kim, Jungyun Lim, Yong-Wook Baek, Sunkyoung Shin, Mina Ha, Hae-Kwan Cheong, Kyu Hyuck Chung, Review Committee for the Epidemiological Correlations between Humidifier Disinfectants Exposure and Health Effects

**Affiliations:** 1 Korea University College of Pharmacy, Sejong, Korea; 2 Sungkyunkwan University, School of Pharmacy, Suwon, Korea; 3 EH R&C Co., Incheon, Korea; 4 Humidifier Disinfectant Health Center, Environmental Health Research Department, National Institute of Environmental Research, Incheon, Korea; 5 Dankook University College of Medicine, Cheonan, Korea; 6 Sungkyunkwan University, School of Medicine, Suwon, Korea; 7 Kyungsung University College of Pharmacy, Busan, Korea

**Keywords:** Evidence-based toxicology, Humidifier disinfectants, Respiratory diseases, Aggregate exposure pathway, Adverse outcome pathway, Biological plausibility

## Abstract

**OBJECTIVES:**

Exposure to humidifier disinfectants has been linked to respiratory diseases, including interstitial lung disease, asthma, and pneumonia. Consequently, numerous toxicological studies have explored respiratory damage as both a necessary and sufficient condition for these diseases. We systematically reviewed and integrated evidence from toxicological studies by applying the evidence integration method established in previous research to confirm the biological plausibility of the association between exposure and disease.

**METHODS:**

We conducted a literature search focusing on polyhexamethylene guanidine phosphate (PHMG) and chloromethylisothiazolinone/methylisothiazolinone (CMIT/MIT), the primary ingredients in humidifier disinfectants. We selected relevant studies based on their quality and the population, exposure, comparator, outcome (PECO) statements. These studies were categorized into three lines of evidence: hazard information, animal studies, and mechanistic studies. Based on a systematic review, we integrated the evidence to develop an aggregate exposure pathway–adverse outcome pathway (AEP-AOP) model for respiratory damage. The reliability and relevance of our findings were assessed by comparing them with the hypothesized pathogenic mechanisms of respiratory diseases.

**RESULTS:**

By integrating toxicological evidence for each component of the AEP-AOP framework for PHMG and CMIT/MIT, we developed an AEP-AOP model that elucidates how disinfectants released from humidifiers expose target sites, triggering molecular initiating events and key events that ultimately lead to respiratory damage. This model exhibits high reliability and relevance to the pathogenesis of respiratory diseases.

**CONCLUSIONS:**

The AEP-AOP model developed in this study provides strong evidence, based on evidence-based toxicology, that exposure to humidifier disinfectants causes respiratory diseases. This model demonstrates the pathways leading to respiratory damage, a hallmark of these conditions.

## GRAPHICAL ABSTRACT

[Fig f5-epih-46-e2024060]

## Key Message

• The AEP-AOP model for humidifier disinfectant-induced respiratory damage developed by the AEP-AOP frameworks for PHMG and CMIT/MIT, along with the toxicological evidence integration method.

• This model provides a scientific basis for establishing causality between mixed exposure to humidifier disinfectants and respiratory diseases, and can be used in the future to predict the health effects of various disinfectant ingredients.

## INTRODUCTION

Since the humidifier disinfectant incident in Korea in 2011, legislation has been implemented to provide compensation to victims. The “Special Act on Remedy for Damage Caused by Humidifier Disinfectant,” revised in September 2020, mandates the confirmation of epidemiological correlations through comprehensive evidence gathered from various investigations and studies, including toxicological research. Therefore, establishing actual causality hinges critically on confirming epidemiological correlation by considering all relevant evidence according to the total evidence approach [1].

Evidence-based toxicology, which integrates toxicological evidence using the weight-of-evidence (WoE) method and is widely used in legal and medical fields, has evolved from this perspective. The National Toxicology Program of the United States has established guidelines for hazard assessment that reflect this comprehensive methodology [2]. Additionally, the Organization for Economic Cooperation and Development (OECD) has issued guidance and outlined key elements for implementing the WoE method in chemical management programs [3]. This process involves documenting the collection and evaluation of evidence, and assigning weights through a stepwise procedure. This procedure includes problem formulation (hypothesis generation), evidence collection (establishing lines of evidence and identifying knowledge gaps), evidence evaluation (assessing reliability and relevance), WoE (evaluating the lines of evidence), and evidence integration and reporting (determining the consistency of evidence).

Dekant et al. [4] developed a quantitative WoE method to assess the reliability of predicted modes of action from animal toxicity studies for classification and labeling purposes. They applied this method in case studies involving methyl tert-butyl ether and octamethyltetracyclosiloxane. Martin et al. [5] highlighted the importance of establishing WoE in a harmonized manner across different fields to consolidate and integrate lines of evidence. Additionally, an objective understanding is enhanced by conducting comprehensive literature searches, exchanging information with relevant agencies, and making expert decisions.

The aggregate exposure pathway (AEP) delineates the routes from a chemical source to the target exposure site. This framework evaluates the pathways by analyzing the characteristics of emissions, environmental concentrations, transport to the body, and potential transformations during transport, ultimately assessing the exposure level at the target site. Specifically, for inhalation exposure, it determines the form and concentration of chemicals in the air, the exposure site within the respiratory system, and the exposure level [6].

The adverse outcome pathway (AOP) delineates the biological pathways that disrupt homeostasis, leading to toxicity. It aims to facilitate an intuitive understanding of the toxic effects induced by chemicals. The AOP framework integrates diverse types of information, ranging from the molecular to the individual level, to predict health hazards in humans and provide biological inferences or hypotheses [6]. Within this framework, all evidence regarding health effects is integrated and assessed to support the AOP of a specific chemical. By comparing this evidence with the known pathological mechanisms of the disease in question (AOP hypothesis), a causal relationship between chemical exposure and disease can be established.

Various toxicological studies have examined the toxic effects and mechanisms related to respiratory damage, including interstitial lung diseases, asthma, and pneumonia, resulting from exposure to humidifier disinfectants. Since individual studies are conducted under specific conditions, they often provide only fragmented experimental evidence, necessitating the integration of these studies for a comprehensive assessment. Additionally, due to anatomical, genetic, and physiological differences, animal studies offer a limited perspective on the complex disease mechanisms that occur in humans. To address the limitations of individual toxicity studies, the AEP-AOP framework has been introduced. This framework connects AEP and AOP to assess the entire body of evidence, providing a new approach to establishing causal relationships between exposure and disease [6].

In this study, we conducted a systematic review and integrated evidence from toxicological studies examining respiratory system damage caused by polyhexamethylene guanidine phosphate (PHMG) and chloromethylisothiazolinone/methylisothiazolinone (CMIT/MIT), the primary components of humidifier disinfectants. Utilizing the AEP-AOP framework, which combines exposure assessment, animal studies, and mechanistic studies, we confirmed the biological plausibility and scientifically demonstrated the causal relationship between exposure to humidifier disinfectants and respiratory diseases.

## MATERIALS AND METHODS

**Figure 1.**
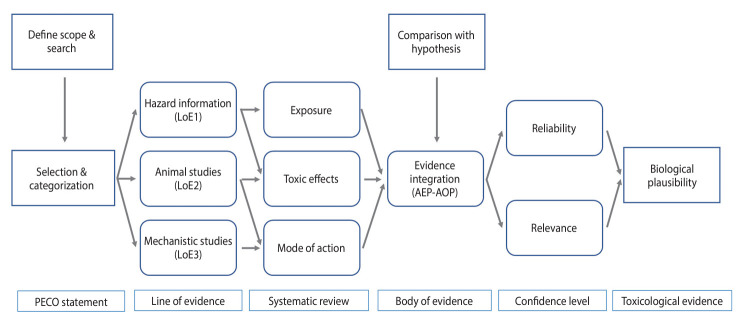
Process for integrating toxicological evidence for biological plausibility between humidifier disinfectant exposure and respiratory disease. AEP-AOP, aggregate exposure pathway-adverse outcome pathway; PECO, population, exposure, comparator, and outcome.

To confirm the biological plausibility of a link between exposure to humidifier disinfectants and respiratory diseases, the toxicological evidence integration method was employed [7]. AEP-AOPs were constructed by integrating evidence from exposure to adverse outcomes (AOs) for PHMG and CMIT/MIT. These were further synthesized to develop an AEP-AOP model for respiratory damage induced by humidifier disinfectants, and its biological plausibility was confirmed by comparing it with the hypothetical mechanisms of respiratory disease (Figure 1).

### Outline and scope of literature review

We defined the outline and scope of the literature search for relevant toxicological studies using population (P), exposure (E), comparator (C), and outcome (O) statements (PECO) as shown in Table 1. The PECO approach is a critical tool for understanding and evaluating the association between chemical exposure and health outcomes by clearly defining the purpose and direction of systematic review and specifying the research design. The literature search, evidence integration, and systematic review were conducted using PECO statements.

### Identification of relevant studies

**Figure 2.**
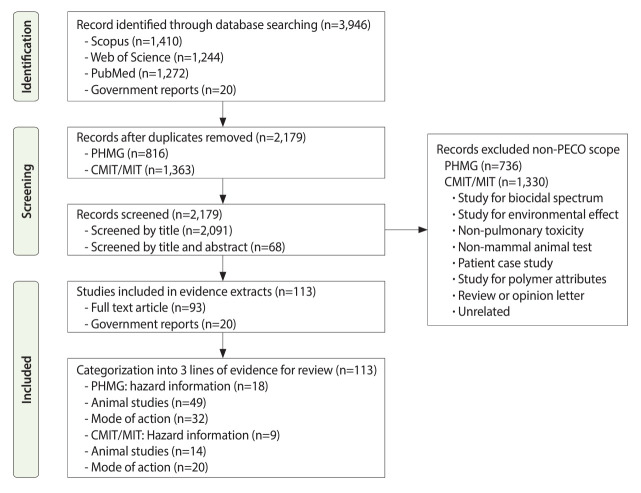
Prisma flowchart for the systematic reviews, which included searching databases related to polyhexamethylene guanidine phosphate (PHMG) and chloromethylisothiazolinone/methylisothiazolinone (CMIT/MIT)-induced respiratory damage. PECO, population, exposure, comparator, and outcome.

To identify toxicological studies on PHMG and CMIT/MIT, the search terms used included “polyhexamethylene guanidine” and “methylchloroisothiazolinone OR methylisothiazolinone OR 5-chloro-2-methyl-4-isothiazolin-3-one OR 2-methyl-4-isothiazolin-3-one.” Studies were evaluated for quality according to the “Toxicological data Reliability Assessment Method”[8], and valid studies were selected. Additionally, the relevance to the PECO scope was assessed to determine the suitability of the data for use as evidence of respiratory damage. After reviewing the original articles, the data were categorized into 3 types of evidence: hazard information, mechanistic studies, and animal studies (Figure 2).

### Review of toxicological literature for lines of evidence

Based on the selected toxicological literature, we extracted evidence concerning exposure, mode of action, and toxic effects. We analyzed the exposure pathway using the AEP framework [6], which delineates how components of disinfectants emitted from humidifiers are inhaled and subsequently deposited at the respiratory target site.

From mechanistic studies, we analyzed the toxicity pathway by which humidifier disinfectants that reach the target site induce disease using the AOP framework [6]. This analysis included the initial reactions with macromolecules (molecular initiating event) and the subsequent biological responses in organelles, cells, tissues, and organs (key events, KEs). Finally, we identified adverse outcome (AO) related to respiratory disease by collecting evidence from animal studies.

### Aggregate exposure pathway-adverse outcome pathway framework and assessment of biological plausibility

This study employed the AEP-AOP framework [6] to integrate three lines of evidence: hazard information, animal studies, and mechanistic studies. This approach enabled the development of AEP-AOPs for PHMG and CMIT/MIT, culminating in a detailed AEP-AOP model of respiratory damage caused by humidifier disinfectants. The model delineated connections among sequential elements including target site exposure, molecular initiating events, KEs, and AOs.

To evaluate the biological plausibility of an association between humidifier disinfectant exposure and respiratory diseases, we compared the AEP-AOP model to the hypothetical pathogenic mechanism outlined in clinical and/or epidemiological studies [1]. The confidence level in the AEP-AOP model was assessed based on established criteria for both reliability (Supplementary Material 1) and relevance (Supplementary Material 2). Reliability pertains to the strength and consistency of the dose-response relationships observed across various studies. We applied the Bradford-Hill criteria to assess whether exposure to humidifier disinfectants consistently led to pulmonary damage across studies and whether the observed toxicity’s strength and dose-response relationship remained consistent. Relevance was evaluated according to the OECD AOP Development and Evaluation Guidelines [3], focusing on the adequacy of the pathway, the essentiality of the KEs, and the sufficiency of data. The adequacy of the pathway was assessed by comparing it to the hypothetical pathogenesis of respiratory diseases, grounded in existing biological knowledge. The essentiality of KEs was evaluated by examining whether downstream KEs are impacted when upstream KEs are modified or prevented. Data sufficiency was determined by assessing whether there is sufficient evidence to support the relationships between KEs. Each factor was rated as “high,” “low,” or “unclassified.” The overall reliability and relevance were then categorized as “high” if the assessment score averaged as “high,” “low” if the average was “low,” and “unclassified” if the average fell between “high” and “low” or if the evidence was insufficient.

### Ethics statement

The study dose not involve human or animal subjects but uses existing literature.

## RESULTS

### Selection of relevant studies and categorization of lines of evidence

A literature search for toxicological studies on PHMG and CMIT/MIT yielded 3,926 publications from databases such as PubMed, Web of Science, and Scopus. We also included 16 domestic and 4 international government reports. After screening titles and abstracts, duplicates were removed (816 for PHMG; 1,369 for CMIT/MIT). Studies that did not meet our defined PECO criteria were excluded (736 for PHMG; 1,330 for CMIT/MIT). Ultimately, 113 studies were selected for the systematic review (Supplementary Material 3).

### Systematic review of aggregate exposure pathway for humidifier disinfectants

PHMG and CMIT/MIT are classified within the guanidine and isothiazolinone families, respectively. PHMG functions as an ionic compound, interacting with both the hydrophilic and lipophilic parts of cell membrane phospholipids, which disrupts the membrane and exhibits bactericidal effects. On the other hand, CMIT/MIT is hydrophilic and dissolves easily in water at room temperature (15-25°C), exhibiting some volatilization. It reacts with thiol groups in proteins, leading to oxidative damage. Due to their physicochemical properties, exposure to these chemicals can result in toxicity [1].

Among the various types of humidifiers, ultrasonic humidifiers utilize ultrasonic vibrations to disperse a humidifying solution from a reservoir into the air as a fine mist. These humidifiers are known to produce mist containing higher concentrations of particulate matter, with predominantly smaller particle sizes (< 2.5 µm) compared to other humidifier types [9]. Consequently, these particles are more likely to reach the alveoli and have a higher deposition rate. The number of particles generated depends on the type of water used in the humidifier, with the order being tap water> filtered water> distilled water. The particle sizes are predominantly below 0.4 µm, making them easily respirable [10]. The presence of humidifier disinfectant particles, approximately 30-60 nm in size, as aerosols in the air has also been reported [11]. Under typical humidifier operating conditions, hydrophilic compounds dissolved in the humidifier water are atomized into the air. During this process, moisture and volatiles contained in small water droplets rapidly evaporate, leaving behind minute aerosols (tens to hundreds of nanometers) suspended in the air. The majority of these particles are distributed in the size range below 5 µm (0.0005 cm), with a peak distribution observed between 0.2-0.4 µm (0.00002-0.00004 cm). As CMIT/MIT is semi-volatile, it may also partially exist in the gas phase [12]. Therefore, both theoretically and experimentally, it has been established that humidifier disinfectant components can emit into the air as aerosols or gases, depending on their physicochemical properties.

The human body can be exposed to humidifier disinfectant components released into the air through various routes, including the respiratory system, skin, ocular mucosa, and oral cavity. However, this study specifically focused on respiratory damage, thus limiting the exposure routes to the respiratory tract alone. The respiratory system, a complex network, extends from the trachea to the alveoli and includes at least 32 branching airways that require small, low-inertia particles to navigate through. Aerosols generated by humidifiers disintegrate into smaller particles, which can easily alter their trajectory due to decreased resistance as pressure drops. During inhalation, external substances are drawn into the respiratory system, and an increase in aerosol concentration in the air leads to more particles reaching the distal lung. Additionally, the movement of aerosols into the lungs is heightened in individuals with elevated respiratory rates, such as children, pregnant women, and patients with respiratory diseases. Using the multi-path particle dosimetry model developed by the Netherlands National Institute for Public Health and the Environment, it was found that humidifier disinfectant aerosols predominantly fall within the 0.2-0.4 µm size range. These particles are capable of reaching the pulmonary periphery, including the alveolar cells, via both the upper and lower airways. Furthermore, the evaluation of individual exposure levels based on the results of a personal exposure assessment survey—which considered factors such as individual product use, duration and frequency of use, and the volume of the usage space—revealed that most reported victims were exposed to levels exceeding the inhalation toxicity reference values (PHMG 0.77 µg/m³, CMIT/MIT 0.127 µg/m³) [1].

### Systematic review of adverse outcome pathway for humidifier disinfectants

Due to its strong cationic properties, PHMG readily adheres to the negatively charged cell membranes of epithelial cells in the respiratory tract and lungs. This adherence disrupts the membrane and compromises cellular homeostasis upon exposure [13,14]. Additionally, PHMG penetrates cells, damaging lysosomes and mitochondria, which leads to cell death [11,14-19]. PHMG also induces inflammation [15,17,18,20,21], with neutrophils playing a pivotal role in this process. Key molecular markers of inflammation, such as Nalp3, interleukin (IL)-1β, tumor necrosis factor-α, IL-6, IL-8, interferon-γ, CCL2 (MCP-1), CXCL1, and prostaglandin E2, are linked to type 1 helper T cell-mediated inflammation. This inflammation triggers changes in extracellular matrix metalloproteinases, which are activated in response to both the induction and resolution of inflammation and facilitate the movement of leukocytes, platelets, and other cells. Changes in molecular markers (matrix metalloprotein [MMP]2, MMP9, MMP12, TIMP1, and TIMP2) associated with these processes have been observed in mechanistic studies [15,17,21,22]. Repeated or excessive exposure to PHMG has been shown to cause excessive deposition of extracellular matrix proteins, such as collagen and fibronectin, leading to airway remodeling [15,21,23-25]. In summary, the mechanisms underlying PHMG-induced respiratory toxicity have been consistently reported.

Table 2 summarizes representative studies that clearly report key endpoints in the systematic review of animal studies on PHMG-induced respiratory damage [26-30]. Tissue destruction caused by PHMG is characterized by airway remodeling, an increase in extracellular matrix, fibroblast foci, and hemorrhaging in the respiratory tract. The respiratory damage from PHMG extends throughout the respiratory tract, from the upper airways to the distal lung. It was consistently and notably observed across different species, strains, and exposure methods, with the severity of histopathological findings increasing proportionally with the exposure dose.

CMIT/MIT reaches the respiratory epithelial cells in the airways and lungs, where it either converts into reactive electrophilic species or enhances the production of reactive oxygen species, initiating toxic interactions with intracellular macromolecules. This compound restricts cell proliferation by inducing cell cycle arrest, which is associated with p53/p21 activation [31]. Additionally, it increases the expression of proteins linked to mitochondrial membrane depolarization, leading to cell death through oxygen deprivation and inhibition of ATP synthesis [32]. Stimulation of epithelial cells triggers the activation of Th2-related signaling molecules (IL-4, IL-5, and IL-13) and causes eosinophilic inflammation [33,34]. It also leads to the secretion of substances such as TSLP, IL-33, and IL-25, which activate ILC2 and Th2 cells [33]. Furthermore, the induction of epithelial stimulation promotes eosinophilic inflammation, which encourages M2 polarization of macrophages and sustained increases in transforming growth factor-β. This results in goblet cell hyperplasia and mucin overproduction, culminating in airway inflammation [32].

Table 2 summarizes the representative studies with clearly reported KEs in the systematic review of animal studies on CMIT/MIT-induced respiratory damage. The extent of pulmonary parenchymal damage varied depending on the species, strain, and route of exposure. While no pulmonary parenchymal damage was observed in studies involving systemic or nasal inhalation exposure [22,26,36,37], significant pulmonary parenchymal damage was noted in studies that used intratracheal instillation [25,26,35]. In the upper airways, inflammation was reported in several studies of systemic inhalation exposure [22,35,37], with moderate-to-severe bronchiolar inflammation observed in a study involving C57BL/6 mice [34]. Intratracheal instillation involves introducing the test substance directly into the respiratory tract, allowing it to reach the lungs through respiration. This method provides a more direct assessment of the local effects in the lungs compared to systemic or non-nasal inhalation exposure. The absence of pulmonary parenchymal damage in studies reporting systemic or non-nasal inhalation exposure may be attributed to differences in dose levels and the anatomical characteristics of the respiratory system in the experimental animals used. Therefore, although the degree of pulmonary parenchymal damage varied with the species and strain of the experimental animals, the method of exposure, and the concentration of the substance, tissue-level observations indicated tissue destruction. This included airway remodeling, fibroblast lesions, and hemorrhage, with the severity of pathological findings increasing with the exposure dose.

### Aggregate exposure pathway-adverse outcome pathway model development and determination of biological plausibility

**Figure 3.**
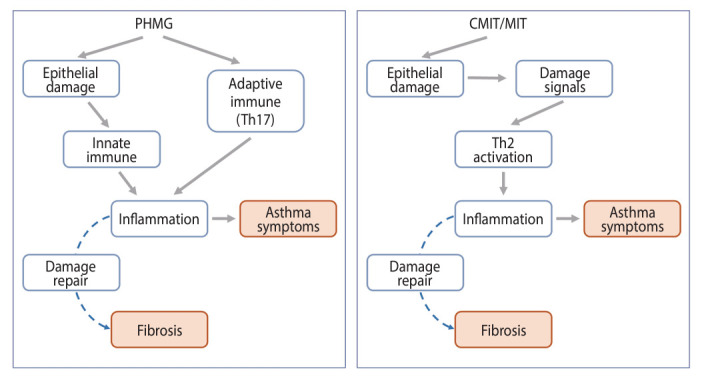
Schematic overview of the mode of action of polyhexamethylene guanidine phosphate (PHMG) and chloromethylisothiazolinone/methylisothiazolinone (CMIT/MIT)-induced respiratory damage.

Figure 3 summarizes the mechanisms of respiratory damage caused by PHMG and CMIT/MIT. While there are some differences in the initial-KEs of the AOP, both agents commonly lead to respiratory diseases, including fibrosis and asthma.

**Figure 4.**
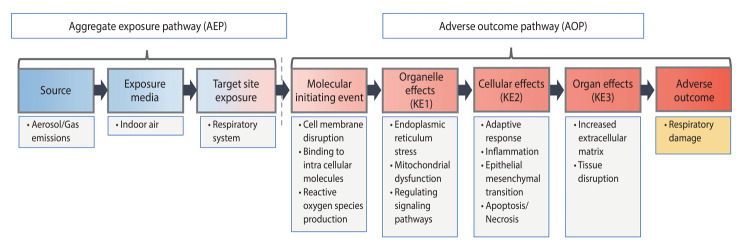
Conceptual diagram for the AEP-AOP model of humidifier disinfectant-induced respiratory damage. KE, key events.

By integrating the entire body of evidence based on the AEP-AOP framework for PHMG and CMIT/MIT, pathways from exposure to AOs were established for each component. The AEP-AOPs constructed for each component were synthesized into a comprehensive model illustrating the respiratory damage caused by humidifier disinfectants (Figure 4). This model depicts the release of aerosols and gaseous components from humidifiers, leading to exposure at the target sites in the respiratory system. It demonstrates that chemical stimulation by direct-acting toxicants initiates a molecular event, which triggers subsequent responses (KE1), such as organelle stress and signaling cascades. These are followed by responses (KE2) that include inflammation, epithelial-mesenchymal transition, and cell death, leading to tissue changes (KE3) characterized by increased extracellular matrix and tissue destruction. These KEs progress sequentially, ultimately resulting in respiratory damage as an AO.

The developed AEP-AOP model demonstrated high reliability when compared with the pathological mechanisms proposed in clinical medicine and the epidemiology of respiratory diseases such as interstitial lung disease, asthma, and pneumonia. Additionally, the strength and dose-response relationships of the toxic responses were consistent across various toxicological studies. The alignment of stepwise elements with pathological mechanisms also showed high relevance. Consequently, the confidence level of the AEP-AOP model for respiratory damage was rated as “high,” confirming a clear biological plausibility between exposure to humidifier disinfectants and respiratory disease.

## DISCUSSION

The amended “Special Act on Remedy for Damage Caused by Humidifier Disinfectant,” as of September 2020, stipulates that confirmation of epidemiologic correlation is required for causal inference [1]. Consequently, a novel approach integrating epidemiological and toxicological evidence was proposed to establish these correlations. In examining the epidemiological link between exposure to humidifier disinfectants and related diseases, clinical and epidemiological evidence indicates that inhaling these disinfectants initially damages the respiratory system. This damage includes epithelial cell apoptosis, the generation of reactive oxygen species, and organelle damage. Such initial harm is believed to lead to various respiratory diseases, including interstitial lung disease, asthma, and pneumonia. Therefore, this respiratory damage can be regarded as both a necessary and sufficient condition for these diseases, as supported by multiple populations and diverse epidemiological study designs. As a result, interstitial lung disease, asthma, and pneumonia are now acknowledged as health hazards covered by the causal inference criteria of the “Special Act on Humidifier Disinfectants.”

When chemical accidents occur, experimental toxicological studies are conducted to establish the relationship between cause and effect. These studies provide data that not only support epidemiological studies but also offer evidence for biological plausibility. While animal studies can bolster epidemiological findings, their results must be interpreted with caution due to interspecies differences between humans and animals. The development of *in vitro* research models that mimic human biological processes has enhanced the relevance to human health and may provide more accurate reflections than animal models [38]. Furthermore, a comprehensive evaluation of the scientific evidence from toxicological studies can lead to objective and transparent conclusions about the causality of health effects. The AEP-AOP framework integrates the entire body of evidence and systematically analyzes the causal relationship between chemical exposure and health effects, offering a comprehensive understanding that individual studies alone cannot provide [39].

Although the KEs that constitute the adverse outcome pathway may vary among chemicals with similar physicochemical properties that induce the same disease, the overall pathway is likely to be very similar in a broader context, such as chemical damage leading to respiratory cell damage, then inflammation, and ultimately respiratory disease [40]. Humidifier disinfectants, primarily used for their antimicrobial properties, share common physicochemical characteristics. The major ingredients in these disinfectants were PHMG and CMIT/MIT, with the most significant damage observed in users of combined products.

Therefore, this study comprehensively identified the biological plausibility of the association between humidifier disinfectant exposure and respiratory disease by integrating the AEP-AOP for PHMG and CMIT/MIT-induced respiratory damage, closely reflecting the actual exposure situation. Further research on humidifier disinfectant components other than PHMG and CMIT/MIT is necessary to refine the AEP-AOP model presented in this study. The AEP-AOP model for humidifier disinfectant-induced respiratory damage and the toxicological evidence integration method developed in this study can be used in the future to predict the respiratory health effects of various disinfectant ingredients.

## Figures and Tables

**Figure f5-epih-46-e2024060:**
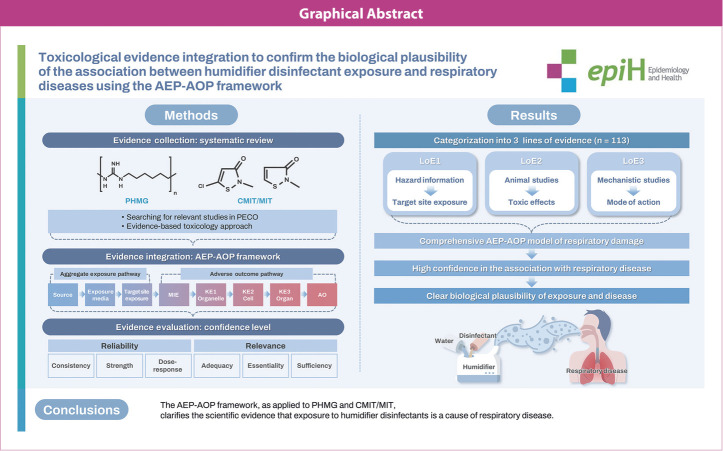


**Table 1.** Description of a PECO for a toxicological systematic review of humidifier disinfectant-induced respiratory damage

PECO	Inclusion criteria
Population (P)	Laboratory animal (species, age, sex)
	Cell line (type, origin, passaging)
Exposure (E)	Characterization of test substance (humidifier disinfectant ingredients)
	Information on administration route (whole body, nasal, intratracheal instillation)
	Doses or concentrations administered and selection basis
	Frequency and duration of exposure
	Experimental conditions for cell culture and chemical treatment
Comparator (C)	Control groups (vehicle, positive, negative)
Outcome (O)	Complete test results, explicit biological effects
	Explanation of results with biological plausibility (histologic findings and biomarkers of respiratory damage)

**Table 2.** Summary of systematic reviews of animal studies on respiratory damage by PHMG and CMIT/MIT

Study	Species	Exposure route	Key events	Relevance
PHMG				
KCDC, 2011 [26], NIER, 2021 [27]	C57BL/6 mouse	Intratracheal instillation	Lung Inflammatory cell foci, increased fibronectin, squamous metaplasia, foamy macrophage aggregates, broncho-alveolar hyperplasia, airway resistance, mucous cell hyperplasia	Confirmed
KCDC, 2011 [26]	SD rat	Whole body inhalation	Inflammatory cell foci, squamous metaplasia	Confirmed
NIER, 2013 [28]	SD rat	Nasal inhalation	Inflammatory cell, foamy macrophage aggregates, epithelial de/regeneration, bronchiole, congestion/hemorrhage, fibrosis	Confirmed
MOE, 2017 [29]	F344 rat	Whole body inhalation	Inflammatory cell infilteration, fibrosis, hyperplasiam hemorrhage	Confirmed
KEI, 2019 [30]	Balb/c mouse	Intratracheal instillation	Airway resistance, inflammatory cytokines, mucous cell hyperplasia, asthma associated cytokines	Confirmed
CMIT/MIT				
NIER, 2021 [27], KEI, 2019 [30]	C57BL/6 mouse	Intratracheal instillation	Asthma associated gene analysis, M2 marker upregulation, airway resistance, mucous cell hyperplasia	Confirmed
NIER, 2018 [35]	C57BL/6 mouse	Whole body inhalation	Acute inflammation, bronchioloalveolar, erosion/ulceration, squamous epithelium	Confirmed
KCDC, 2011 [26]	C57BL/6 mouse	Intratracheal instillation	Inflammation, histiocytes, alveolar, multinucleated giant cell, fibrosis, broncho-alveolar hyperplasia	Confirmed
KCDC, 2011 [26]	SD rat	Whole body inhalation	No specific toxic reactions	Unconfirmed
KIT, 2022 [36]	SD rat	Nasal inhalation	No specific toxic reactions	Unconfirmed
KEI, 2018 [22]	SD rat	Whole body inhalation	(Olfactory) epithelial atrophy/degeneration, inflammatory cell infiltration	Partially confirmed (upper respiratory tract)
KEI, 2018 [22]	SD rat	Nasal inhalation	Inflammatory cell infiltration	Unconfirmed
Song et al., 2020 [37]	Wistar rat	Whole body inhalation	(Olfactory) epithelial atrophy/degeneration, transitional epithelial degeneration/regeneration, and/or inflammatory cell infiltration, epithelial hyperplasia, squamous metaplasia	Partially confirmed (upper respiratory tract)

PHMG, polyhexamethylene guanidine phosphate; CMIT/MIT, chloromethylisothiazolinone/methylisothiazolinone; KCDC, Korea Centers for Disease Control and Prevention; NIER, National Institute of Environmental Research; MoE, Ministry of Environment; KEI, Korea Environmental Industry & Technology Institute; KIT, Korea Institute of Toxicology.

## Supplementary materials

Korean version is available at https://doi.org/10.4178/epih.e2024060.

Supplementary Material 1. Reliability evaluation criteria for AEP-AOP of humidifier disinfectant-induced respiratory damage

Supplementary Material 2. Relevance evaluation criteria for AEP-AOP of humidifier disinfectant-induced respiratory damage

Supplementary Material 3. Literature list included in the systematic review
