# Supplementary material for: Toxicological evidence integration to confirm the biological plausibility of the association between humidifier disinfectant exposure and respiratory diseases using the AEP-AOP framework
Source: Epidemiol Health. 2024 Jul 7;46:e2024060. doi: 10.4178/epih.e2024060 (PMC11576529; doi:10.4178/epih.e2024060)
Supplement: Supplementary Material 1. — Reliability evaluation criteria for AEP-AOP of humidifier disinfectant-induced respiratory damage [file epih-46-e2024060-Supplementary-1.docx]

Supplementary Material 1. Reliability evaluation criteria for AEP-AOP of humidifier disinfectant-induced respiratory damage

| **Criteria** | **Question** | **High** | **Low** | **Unclassifiable** |
| --- | --- | --- | --- | --- |
| Consistency | Are results on respiratory damage consistent across study designs? | Results from extensive studies are mostly consistent. | There is some variability or conflicting results between studies | Consistency cannot be determined due to limited number or diversity of studies |
| Strength | Are there significant changes associated with respiratory damage? | Respiratory damage-related toxicity are statistically significant | Respiratory damage-related toxicity are not statistically significant | Insufficient studies to determine statistical significance |
| Dose-response | Is there a dose-response relationship for respiratory damage-related toxicity? | A clear dose-response is observed | A dose-response is observed, but not as clear or consistent | Insufficient studies to establish dose-response relationship |
