# Supplementary material for: Toxicological evidence integration to confirm the biological plausibility of the association between humidifier disinfectant exposure and respiratory diseases using the AEP-AOP framework
Source: Epidemiol Health. 2024 Jul 7;46:e2024060. doi: 10.4178/epih.e2024060 (PMC11576529; doi:10.4178/epih.e2024060)
Supplement: Supplementary Material 2. — Relevance evaluation criteria for AEP-AOP of humidifier disinfectant-induced respiratory damage [file epih-46-e2024060-Supplementary-2.docx]

Supplementary Material 2. Relevance evaluation criteria for AEP-AOP of humidifier disinfectant-induced respiratory damage

| **Criteria** | **Question** | **High** | **Low** | **Unclassifiable** |
| --- | --- | --- | --- | --- |
| Adequacy | Is the AEP-AOP* consistent with the established biological knowledge of respiratory diseases? | Broad acceptance and scientific understanding is completely established | Analogy to the knowledge but scientific understanding is not completely established | Unable to configure the AEP-AOP due to lack of research |
| Essentiality | Is there an impact on downstream KEs if upstream KE is modified or prevented? | Specifically designed experimental evidence showing prevention or impact | No or contradictory experimental evidence of the essentiality of any of the KEs | Limited or no studies reporting the essentiality of the KEs |
| Sufficiency | Is there sufficient evidence to support a causal relationship between KEs? | Multiple studies showing dependent changes in both upstream and downstream KEs | Few studies showing dependent change in both KEs and some evidence inconsistent with expected pattern | Limited or no studies reporting dependent change in both KEs |

*AEP-AOP: Aggregate Exposure Pathway-Adverse Outcome Pathway
