# Supplementary material for: Toxicological evidence integration to confirm the biological plausibility of the association between humidifier disinfectant exposure and respiratory diseases using the AEP-AOP framework
Source: Epidemiol Health. 2024 Jul 7;46:e2024060. doi: 10.4178/epih.e2024060 (PMC11576529; doi:10.4178/epih.e2024060)
Supplement: Supplementary Material 3. — Literature list included in the systematic review [file epih-46-e2024060-Supplementary-3.docx]

Supplementary Material 3. Literature list included in the systematic review

| **Title** | **Journal** | **Authors** | **Key events** |
| --- | --- | --- | --- |
| 90-Day Repeated Inhalation Toxicity of PHMG-HCL in F344 Rats | Government report (2017) | NA | inflammatory cell infilteration, fibrosis, hyperplasiam hemorrhage, |
| A comprehensive study of deaths due to exposure to humidifier disinfectant in Korea: focusing on medical records, assessment of exposure to humidifier disinfectants, and causes of death. | Epidemiology and Health (2021) | Ju et al. | Estimate exposure of PHMG-p |
| A humidifier disinfectant biocide, polyhexamethylene guanidine phosphate, inhalation exposure during pregnancy induced toxicities in rats. | Journal of Hazardous Materials (2021) | Lee et al. | intrauterine growth retardation of fetuses |
| A mixture of chloromethylisothiazolinone and methylisothiazolinone impairs rat vascular smooth muscle by depleting thiols and thereby elevating cytosolic Zn(2+) and generating reactive oxygen species. | Archives of Toxicology (2021) | Quan do et al. | ROS stress, impaired smooth muscle contraction, elevated intracellular Zn2+, depleted thiols |
| A multiplex inhalation platform to model in situ like aerosol delivery in a breathing lung-on-chip. | Frontiers in Pharmocology (2023) | Sengupta et al. | Cytotoxicity, inflammation |
| A safety study for management of the existing chemicals (I) | Government report (2013) | NA | inflammatory cell, Foamy macrophage aggregates,Epithelial de/regeneration, bronchiole, Congestion/hemorrhage, fibrosis |
| A Study on Health Effects of Toxic Chemical Contained in Household Chemical Products (I) | Government report (2014) | NA | Discoloration, Increased alveolar macrophages, pigmented, Inflammatory cell infiltration, Pulmonary fibrosis, Degeneration/regeneration, bronchiolar epithelium),Suppurative exudate |
| A Study on Health Effects of Toxic Chemical Contained in Household Chemical Products (II) | Government report (2016) | NA | No specific toxic reactions |
| Acute cardiovascular toxicity of sterilizers, PHMG, and PGH: severe inflammation in human cells and heart failure in zebrafish. | Cardiovascular Toxicology (2012) | Kim et al. | atherogenic process,Lipoprotein Modification |
| Adverse postnatal developmental effects in offspring from humidifier disinfectant biocide inhaled pregnant rats. | Chemosphere (2022) | Lee et al. | increased perinatal death,decreased postnatal survival after birth,decreased the body weight of offspring after birth, increased the gestation period |
| Aesculetin Attenuates Alveolar Injury and Fibrosis Induced by Close Contact of Alveolar Epithelial Cells with Blood-Derived Macrophages via IL-8 Signaling. | International Journal of Molecular Sciences (2020) | Oh et al. | recruitment of inflammatory cells, increase dense of bronchioles and alveoli |
| Akt and Notch pathways mediate polyhexamethylene guanidine phosphate-induced epithelial-mesenchymal transition via ZEB2. | Pharmacology (2019) | Jeong et al. | Epithelial mesenchymal transition |
| An in vivo study to determine the relationship between humidifier disinfectant and respiratory disease induction and exacerbation | Government report (2019) | NA | Airway resistance, inflammatory cytokines, mucous cell hyperplasia, asthma associated cytokines |
| Analysis of genomic responses in a rat lung model treated with a humidifier sterilizer containing polyhexamethyleneguanidine phosphate. | Toxicology Letters (2017) | Kim et al. | inflammatory response,oxidative stress, |
| Analysis of lung cancer-related genetic changes in long-term and low-dose polyhexamethylene guanidine phosphate (PHMG-p) treated human pulmonary alveolar epithelial cells. | BMC Pharmacology and Toxicology (2022) | Lee et al. | increase lung cancer-associated genes |
| Anti-fibrotic effect of pycnogenol® in a polyhexamethylene guanidine-treated mouse model. | Respiratory Physiology & Neurobiology (2022) | Park et al. | ,lung wall thickens, ECM production, deposition |
| Assessment of acute and repeated pulmonary toxicities of oligo(2-(2-ethoxy)ethoxyethyl guanidium chloride in mice. | Toxicological Research (2017) | Song et al. | increased cytokine production, immune cell infiltration, pulmonary fibrotic changes |
| Assessment of agonistic and antagonistic properties of humidifier disinfectants to the estrogenic and androgenic receptors by transactivation assay. | Toxicological Research (2022) | Lee et al. | estrogenic and androgenic receptors activation |
| Association between use of humidifier disinfectant and allergic rhinitis in Korean children: a cross-sectional study based on the eighth Panel Study on Korean Children (PSKC). | Annals of Occupational and Environmental Medicine (2020) | Koh et al. | allergic rhinitis |
| Association of high-level humidifier disinfectant exposure with lung injury in preschool children. | Science of The Total Environment (2018) | Park et al. | lung injury |
| Biodistribution and respiratory toxicity of chloromethylisothiazolinone/ methylisothiazolinone following intranasal and intratracheal administration. | Environment International (2022) | Song et al. | Biodisrtibution, alveolar granulomatous inflammation/fibrosis and bronchiole mucous cell hyperplasia,erosions and ulcerations in the nasal cavity |
| Causal relationship between humidifier disinfectant exposure and Th17-mediated airway inflammation and hyperresponsiveness. | Toxicology (2021) | Song et al. | higher peribronchial/perivascular inflammation, elevated goblet cell hyperplasia, and inhaled methacholine-induced airway resistance. |
| Changes in expression of cytokines in polyhexamethylene guanidine induced lung fibrosis in mice: Comparison of bleomycin-induced lung fibrosis. | Toxicology (2018) | Kim et al. | nflammatory cell infiltration, fibrosis mainly in the terminal bronchioles and alveoli, increase various cytokines |
| Characteristics of Exposure to Chloromethylisothiazolinone (CMIT) and Methylisothiazolinone (MIT) among Humidifier Disinfectant-Associated Lung Injury (HDLI) Patients in South Korea. | Molecules (2020) | Park et al. | Estimate exposure of CMIT/MIT |
| Characteristics of the Molecular Weight of Polyhexamethylene Guanidine (PHMG) Used as a Household Humidifier Disinfectant. | Molecules (2021) | Park et al. | Estimate exposure characteristics of PHMG-p |
| Chemical allergens stimulate human epidermal keratinocytes to produce lymphangiogenic vascular endothelial growth factor. | Toxicology and Applied Pharmacology (2015) | Bae et al. | VEGF, IL-8 production |
| Classification and characterization of exposure rating in humidifier disinfectants through calculation of PHMG reference concentration. | Journal of Environmental Health Sciences (2020) | Kim et al. | Estimate exposure of PHMG-p |
| CMIT/MIT induce apoptosis and inflammation in alveolar epithelial cells through p38/JNK/ERK1/2 signaling pathway. | Molecular & Cellular Toxicology (2019) | Lee et al. | p38-JNK1/2-ERK1/2 signaling, inflammation, apoptosis |
| Comparative toxicity of polyhexamethylene guanidine phosphate in three strains of rats. | Molecular & Cellular Toxicology (2022) | Woo et al. | granulomatous inflammation, interstitial inflammation, squamous cell metaplasia, and macrophage infiltration in the alveoli |
| Cytotoxicity and gene expression profiling of polyhexamethylene guanidine hydrochloride in human alveolar A549 cells. | Toxicology in Vitro (2014) | Jung et al. | apoptosis, autophagy, fibrosis, cell cycle |
| Development of inhalation toxicity evaluation and attribution techniques for identifying humidifier disinfectants damage | Government report (2018) | NA | inflammatory cell infilteration, fibronectin, CTGF increase, EMT response , Nf-kB signaling |
| Development of technology for hazard assessment of bioside active ingredients | Government report (2015) | NA | bronchioloalveolar hyperplasia, squamous metaplasia, pulmonary fibrosis, inflammatory cell infiltration, foamy macrophage aggregates, degeneration/regeneration, bronchiolar epithelium |
| Development of toxicological indicator discovery technology to identify disease-specific (respiratory and non-respiratory diseases) causality by humidifier disinfectant ingredients | Government report (2020) | NA | inflammatory cytokine, Tight junction decrease, apoptosis, ROS stress |
| Different regulation of T helper 1- and T helper 2-promoting cytokine signalling factors in human dendritic cells after exposure to protein versus contact allergens. | Immunology (2008) | Böttcher et al. | SOCS1, SOCS3, GATA3 expression |
| Disruption of Membrane Integrity as a Molecular Initiating Event Determines the Toxicity of Polyhexamethylene Guanidine Phosphate Depending on the Routes of Exposure. | International Journal of Molecular Sciences (2022) | Song et al. | Increased total BAL cell count, proinflammatory cytokine production, fibrotic changes, |
| Dramatic reduction of toxicity of Poly(hexamethylene guanidine) disinfectant by charge neutralization. | Environmental Research (2023) | Madhappan et al. | Toxic effects of the charge in PHMG-p |
| Effects of 5-chloro-2-methyl-4-isothiazolin-3-one and other candidate biodiesel biocides on rat alveolar macrophages and NR8383 cells. | Archives of Toxicology (2011) | Poon et al. | inhibited phagocytic oxidative burst |
| Effects of lipid membrane composition on the distribution of biocidal guanidine oligomer with solid supported lipid membranes. | RSC Advances (2020) | Yeonjeong Ha, Jung-Hwan Kwon | distribution constant |
| Effects of stabilizer magnesium nirate on CMIT/MIT-induced respiratory toxicity. | Toxicological Research (2023) | Song et al. | increase in inflammatory cell levels in the bronchoalveolar lavage, granulomatous inflammation, mixed inflammatory cell infiltration, mucous cell hyperplasia, eosinophil infiltration, and pulmonary fibrosis |
| Establishment of mouse model for pulmonary inflammation and fibrosis by intratracheal instillation of polyhexamethyleneguanidine phosphate | Journal of Toxicologic Pathology (2016) | Lee et al. | collagen deposition,TGF-β production |
| Estimating retrospective exposure of household humidifier disinfectants. | Indoor Air (2015) | Park et al. | Estimate exposure of PHMG, CMIT/MIT |
| Evaluating Health Effects Investigations of Humidifier Disinfectants for Exposure Characteristics | Government report (2021) | NA | asthma associated gene analysis, MAPK singnaling |
| Evaluating the comparative MT1B, MT1F, MT1G, and MT1H expression in human pulmonary alveolar epithelial cells treated with polyhexamethylene guanidine-phosphate, chloromethylisothiazolinone/methylisothiazolinone, oligo(2-(2-ethoxy)ethoxyethyl guanidinium chloride, benzalkonium chloride, and sodium dichloroisocyanurate | Molecular & Cellular Toxicology (2023) | Kim et al. | MT1B, MT1F, MT1G,MT1H expression increase |
| Evaluation of polyhexamethylene guanidine-induced lung injuries by chest CT, pathologic examination, and RNA sequencing in a rat model. | Scientific Reports (2021) | Kim et al. | causes lung tumors |
| Evaluation of the long-term effect of polyhexamethylene guanidine phosphate in a rat lung model using conventional chest computed tomography with histopathologic analysis. | PLOS ONE (2021) | Kim et al. | causes lung tumors |
| Experimental determination of indoor air concentration of 5-chloro-2-methylisothiazol-3(2H)-one/ 2-methylisothiazol-3(2H)-one (CMIT/MIT) emitted by the use of humidifier disinfectant. | Environmental Analysis Health and Toxicology (2020) | Park et al. | Estimate exposure of CMIT/MIT |
| Exposure characteristics of familial cases of lung injury associated with the use of humidifier disinfectants. | Environmental Health (2014) | Park et al. | Estimate exposure of PHMG |
| Exposure to cigarette smoke exacerbates polyhexamethylene guanidine-induced lung fibrosis in mice. | The Journal of Toxicological Sciences (2021) | Shin et al. | increase number of inflammatory cell, granulomatous inflammation, fibrosis |
| Exposure to Polyhexamethylene Guanidine Exacerbates Bronchial Hyperresponsiveness and Lung Inflammation in a Mouse Model of Ovalbumin-Induced Asthma. | Allergy, Asthma & Immunology Research (2021) | Lee et al. | enhance allergic responses in asthma model |
| Family-based case-control study of exposure to household humidifier disinfectants and risk of idiopathic interstitial pneumonia. | PLOS ONE (2019) | Lamichhane et al. | Estimate exposure of PHMG,CMIT/MIT |
| Fibrinogen on extracellular vesicles derived from polyhexamethylene guanidine phosphate-exposed mice induces inflammatory effects via integrin beta. | Ecotoxicology and Environmental Safety (2023) | Kim et al. | increase sustained inflammation |
| Final Report on the Safety Assessment of Methylisothiazolinone and Methylchloroisothiazolinone | Government report (1992) | NA | present various animal RfD |
| Functional and dynamic mitochondrial damage by chloromethylisothiazolinone/methylisothiazolinone (CMIT/MIT) mixture in brain endothelial cell lines and rat cerebrovascular endothelium. | Toxicology Letters (2022) | Kim et al. | Oxidative stress, Mitochondrial morphological and dynamic change, Mitochondrial functional impairment |
| Gene expression related to lung cancer altered by PHMG-p treatment in PBTE cells. | Molecular & Cellular Toxicology (2023) | Park et al. | altered lung cancer associated genes |
| GSH depletion, protein S-glutathionylation and mitochondrial transmembrane potential hyperpolarization are early events in initiation of cell death induced by a mixture of isothiazolinones in HL60 cells. | Biochimica et Biophysica Acta (BBA) - Molecular Cell Research (2006) | Stefano et al. | Oxidative stress, Mitochondrial damage increase |
| Guanidine-based disinfectants, polyhexamethylene guanidine-phosphate (PHMG-P), polyhexamethylene biguanide (PHMB), and oligo(2-(2-ethoxy)ethoxyethyl guanidinium chloride (PGH) induced epithelial-mesenchymal transition in A549 alveolar epithelial cells. | Inhalation Toxicology (2019) | Park et al. | Epithelial mesenchymal transition |
| Hematotoxic Effect of Respiratory Exposure to PHMG-p and Its Integrated Genetic Analysis. | Toxics (2022) | Sung et al. | increasing hemoglobin and hematocrit levels in peripheral blood,reduced number of megakaryocytes |
| Human exposure to polyhexamethylene guanidine phosphate from humidifiers in residential settings: Cause of serious lung disease. | Toxicology and Industrial settings (2017) | Lee et al. | Estimate exposure of PHMG |
| Humidifier disinfectant and use characteristics associated with lung injury in Korea. | Indoor Air (2019) | Ryu et al. | use characteristic of PHMG, CMIT/MIT |
| Humidifier Disinfectant Lung Disease Epidemiology White Paper | Government report (2015) | NA | Estimate exposure of CMIT/MIT |
| Humidifier Disinfectant–associated Interstitial Lung Disease in an Animal Model Induced by Polyhexamethylene Guanidine Aerosol | American Journal of Respiratory and Critical Care Medicine (2014) | Park et al. | glass opacities involving the entire lungs, bronchiolocentric destruction with inflammation and fibrosis |
| Identification of mechanisms of pulmonary disease development and evaluation of non-pulmonary health effects (including fetal animal models) of hazardous ingredients (PHMGs and PGHs) in disinfectants | Government report (2019) | NA | Increased gestational age, Increased neonatal deaths, fewer newborns alive  Decreased neonate survival, EMT, fiborsis associated gene expression change |
| Immunohistochemical characterization of oxidative stress in the lungs of rats exposed to the humidifier disinfectant polyhexamethylene guanidine hydrochloride. | Journal of Toxicologic Pathology (2019) | Lee et al. | lveolar/interstitial fibrosis with inflammatory cell infiltration, bronchioalveolar hyperplasia, bronchiolar/alveolar squamous metaplasia, bronchial/bronchiolar epithelial detachment, and alveolar hemorrhage |
| In vitro induction of apoptosis vs. necrosis by widely used preservatives: 2-phenoxyethanol, a mixture of isothiazolinones, imidazolidinyl urea and 1,2-pentanediol. | Biochemical Pharmacology (2002) | Anselmi et al. | Apoptosis |
| in vitro neurotoxicity of methylisothiazolinone, a commonly used industrial and household biocide, proceeds via a zinc and extracellular signal-regulated kinase mitogen-activated protein kinase-dependent pathway | Journal of Neuroscience (2002) | Du et al. | Apoptosis, ERK signaling |
| Inhalation study for risk factor related to mysterious lung disease | Government report (2011) | NA | lung Inflammatory cell foci, increased fibronectin, squamous metaplasia, foamy macrophage aggregates, broncho-alveolar hyperplasia |
| Inhalation toxicity of polyhexamethylene guanidine-phosphate in rats: A 4-week inhalation exposure and 24-week recovery period study. | Chemosphere (2023) | Yang et al. | alveolar macrophages, chronic inflammation, squamous metaplasia, alveolar emphysema, and pulmonary fibrosis |
| Inhaled Kathon may induce eosinophilia-mediated disease in the lung | Environmental Toxicology (2020) | Park et al. | Apoptosis, Th-type cytokines increase, inflammatory cell infilteration |
| Integration of transcriptomics, proteomics and metabolomics identifies biomarkers for pulmonary injury by polyhexamethylene guanidine phosphate (PHMG-p), a humidifier disinfectant, in rats. | Archives of Toxicology (2020) | Lee et al. | Pulmonary fibrosis, chronic inflammation, bronchiol–alveolar fibrosis, and metaplasia of squamous cell,inflammatory response, response to stress, and immune response |
| Intra-tracheal Administration of the Disinfectant Chloromethylisothiazolinone/methylisothiazolinone (CMIT/MIT) in a Pregnant Mouse Model for Evaluating Causal Association with Stillbirth | Journal of Environmental Health Sciences (2018) | Kang et al. | stillbirth rates |
| JAK/STAT pathways are not involved in the direct activation of antigen-presenting cells by contact sensitizers. | Archives of Dermatological Research (2002) | Valk et al. | JAK/STAT pathways |
| Kathon CG Preservatives: An observational study of respiratory damage following whole-body inhalation exposure | Government report (2022) | NA | acute inflammation, bronchioloalveolar, erosion/ulceration, squamous epithelium |
| Kathon Induces Fibrotic Inflammation in Lungs: The First Animal Study Revealing a Causal Relationship between Humidifier Disinfectant Exposure and Eosinophil and Th2-Mediated Fibrosis Induction | Molecules (2020) | Song et al. | inflammatory cell infiltration,pulmonary fibrosis, Th2 associated gene expression |
| Liposome leakage and increased cellular permeability induced by guanidine-based oligomers: effects of liposome composition on liposome leakage and human lung epithelial barrier permeability. | RSC Advances (2021) | Ha et al. | lung epithelial barrier permeability |
| Lung fibroblasts may play an important role in clearing apoptotic bodies of bronchial epithelial cells generated by exposure to PHMG-P-containing solution. | Toxicoloty Letters (2018) | Park et al. | apoptosis, autophagy, enhenced membrane and DNA damage-related proteins |
| Metabolomic study on bleomycin and polyhexamethylene guanidine phosphate-induced pulmonary fibrosis mice models. | Metabolomics (2019) | Seo et al. | Various metabolomic changes |
| Methylisothiazolinone induces apoptotic cell death via matrix metalloproteinase activation in human bronchial epithelial cells. | Toxicoloty in Vitro (2020) | Park et al. | proinflammatory cytokine increase, Apoptosis, ROS stress |
| New-Onset and Exacerbation of Lung Diseases after Short-Term Exposures to Humidifier Disinfectant during Hospitalization. | Toxics (2022) | Lee et al. | Estimate exposure of PHMG |
| NOTCH1 Pathway is Involved in Polyhexamethylene Guanidine-Induced Humidifier Disinfectant Lung Injuries. | Yonsei Medical Journal (2020) | Lee et al. | NOTCH1 expression change |
| Oleanolic acid acetate attenuates polyhexamethylene guanidine phosphate-induced pulmonary inflammation and fibrosis in mice. | Respiratory Physiology & Neurobiology (2018) | Kim et al. | inflammatory cell infiltration, fibrosis in the terminal bronchioles and alveoli, increased alveolar macrophages and bronchioloalveolar epithelial hyperplasia |
| OPINION ON the mixture of 5-chloro-2-methylisothiazolin-3(2H)-one and 2-methylisothiazolin-3(2H)-one | Government report (2009) | NA | present various animal RfD |
| opionion on the safety of poly(hexamethylene) biguanide hydrochloride | Government report (2014) | NA | Estimate exposure of PHMG-p |
| Physical analysis reveals distinct responses of human bronchial epithelial cells to guanidine and isothiazolinone biocides. | Toxicology and Applied Pharmacology (2021) | Kwon et al. | cell area, nuclear area, and nuclear shape, mechano-stress |
| Polyhexamethylene guanidine aerosol triggers pulmonary fibrosis concomitant with elevated surface tension via inhibiting pulmonary surfactant. | Journal of Hazardous Materials (2021) | Li et al. | congestion of capillaries, shortening of cilia, inflammatory cell infiltration into the terminal bronchioles,exfoliated epithelial cells in the bronchioles and alveoli, alveolar atrophy, accumulation of foamy macrophages in the alveolar space, bronchioloalveolar epithelial hyperplasia and fibrosis,elevated surface tension, surfactant protein docking |
| Polyhexamethylene guanidine phosphate aerosol particles induce pulmonary inflammatory and fibrotic responses. | Archives of Toxicology (2016) | Kim et al. | reactive oxygen species (ROS) generation, airway barrier injuries and inflammatory and fibrotic responses |
| Polyhexamethylene Guanidine Phosphate Damages Tight Junctions and the F-Actin Architecture by Activating Calpain-1 via the P2RX7/Ca(2+) Signaling Pathway. | Cells (2019) | Jin et al. | decease tight junction |
| Polyhexamethylene guanidine phosphate increases stress granule formation in human 3D lung organoids under respiratory syncytial virus infection. | Ecotoxicology and Environmental Safety (2022) | Choi et al. | increase stress granule |
| Polyhexamethylene Guanidine Phosphate Induces Apoptosis through Endoplasmic Reticulum Stress in Lung Epithelial Cells. | International Journal of Molecular Sciences (2021) | Jeong et al. | ER stress, apoptosis |
| Polyhexamethylene guanidine phosphate induces IL-6 and TNF-alpha expression through JNK-dependent pathway in human lung epithelial cells. | The Journal of Toxicological Sciences (2018) | Kim et al. | inflammatory cytokine, ERK-JNK signaling |
| Polyhexamethylene guanidine phosphate, chloromethylisothiazolinone, and particulate matter are dispensable for stress granule formation in human airway epithelial cells. | Animal Cells and Systems (2021) | Cambronero-urena et al. | increase stress granule formation |
| Polyhexamethylene guanidine phosphate-induced necrosis may be linked to pulmonary fibrosis. | Toxicology Letters (2022) | Kang et al. | necrosis, ROS stress, inflammation,Fibrinous exudate, Granulomatous inflammation, fibrosis, Alveolar bronchiolization |
| Polyhexamethylene guanidine phosphate-induced ROS-mediated DNA damage caused cell cycle arrest and apoptosis in lung epithelial cells. | The Journal of Toxicological Sciences (2019) | Park et al. | ROS stress, cell cycle inhibition, apoptosis |
| Polyhexamethylene guanidine phosphate-induced upregulation of MUC5AC via activation of the TLR-p38 MAPK and JNK axis. | Chemico-Biological Interactions (2019) | Jeong et al. | mucus hypersecretion, p38-JNK signaling |
| Polyhexamethyleneguanidine phosphate induces cytotoxicity through disruption of membrane integrity. | Toxicology (2019) | Song et al. | ROS stress, apoptosis, cell membrane disruption, |
| Polyhexamethyleneguanidine phosphate induces severe lung inflammation, fibrosis, and thymic atrophy. | Food and Chemical Toxicology (2014) | Song et al. | Hyperplastic epithelial cell, Inflammatory cell foci, reduction in the size of the thymus or spleen |
| Properties of Polyhexamethylene Guanidine (PHMG) Associated with Fatal Lung Injury in Korea. | Molecules (2020) | Park et al. | Estimate exposure of PHMG |
| Protective Effects of Nintedanib against Polyhexamethylene Guanidine Phosphate-Induced Lung Fibrosis in Mice. | Molecules (2018) | Kim et al. | lung injury,inflammatory cells in the bronchoalveolar lavage fluid, bronchioloalveolar epithelial hyperplasia, granulomatous inflammation |
| Rate of humidifier and humidifier disinfectant usage in Korean children: A nationwide epidemiologic study. | Environmental Research (2017) | Yoon et al. | Estimate exposure of PHMG |
| Refined exposure assessment for three active ingredients of humidifier disinfectants. | Environmental Engineering Research (2013) | Lee et al. | Estimate exposure of PHMG,CMIT/MIT |
| Reprogrammed lung epithelial cells by decrease of miR-451a in extracellular vesicles contribute to aggravation of pulmonary fibrosis. | Cell Biology and Toxicology (2022) | Jeong et al. | lung fibrosis, Epithelial mesenchymal transition, Akt signaling |
| Reregistration Eligibility Decision (RED) Methylisothiazolinone | Government report (1998) | NA | Vorious Rfd of CMIT/MIT |
| Risk Factors for Unexplained Lung Injury Cytotoxicity Testing and Human Exposure replication study | Government report (2011) | NA | ROS stress,Estimate exposure of CMIT/MIT |
| Role of intracellular calcium and S-glutathionylation in cell death induced by a mixture of isothiazolinones in HL60 cells. | Biochimica et Biophysica Acta (BBA) - Molecular Cell Research (2009) | Stefano et al. | Oxidative stress, Mitochondrial damage increase, ER stress |
| TGF beta/Smad mediated the polyhexamethyleneguanide areosol-induced irreversible pulmonary fibrosis in subchronic inhalation exposure. | Inhalation Toxicology (2020) | Zhu et al. | upregulation transforming growth factor-beta1 and extracellular matrix remodeling markers, increase neutrophils and lymphocytes, increase collagen deposition |
| The Anti-Fibrotic Effects of CG-745, an HDAC Inhibitor, in Bleomycin and PHMG-Induced Mouse Models. | Molecules (2019) | Kim et al. | increased collagen levels, infiltration of inflammatory cells, Epithelial mesenchymal transition marker increase |
| The role of NF-kappa B signaling pathway in polyhexamethylene guanidine phosphate induced inflammatory response in mouse macrophage RAW264.7 cells. | Toxicology Letters (2015) | Kim et al. | proinflammatory cytokine increase, ROS stress increase, Nf-kB signaling increase |
| Time course of polyhexamethyleneguanidine phosphate-induced lung inflammation and fibrosis in mice. | Toxicology and Applied Pharmacology (2018) | Song et al. | increased Immune cell infiltration, increased collagen deposition in the peribronchiolar and interstitial areas of the lungs, increased fibroblast proliferation, hyperplasia of type II epithelial cells |
| Time-course transcriptomic alterations reflect the pathophysiology of polyhexamethylene guanidine phosphate-induced lung injury in rats. | Inhalation Toxicology (2019) | Song et al. | initial inflammatory responses gene expression, chronic lung fibrosis associated gene expression |
| Toxicity of humidifier disinfectant polyhexamethylene guanidine hydrochloride by two-week whole body-inhalation exposure in rats. | Journal of toxicologic pathology (2020) | Lee et al. | degeneration, atrophy, ulcer, inflammatory cell infiltration, inflammation, and fibrosis in nasal cavity, larynx, trachea, and lungs, atrophy of the spleen, thymus, and reproductive organs |
| Toxicity testing to determine health effects of humidifier disinfectants (PHMG and CMIT/MIT combined use, etc.) | Government report (2018) | NA | Olfactory epithelial atrophy/degeneration, transitional epithelial degeneration/regeneration, and/or inflammatory cell infiltration, Epithelial Hyperplasia, Squamous Metaplasia |
| Transcriptomic Analysis of Polyhexamethyleneguanidine-Induced Lung Injury in Mice after a Long-Term Recovery. | Toxics (2021) | Song et al. | Hyperplasia of type II epithelial cells and bronchiolization, accumulated multifocally in the perivascular connective tissue, Macrophage Polarization |
| Types of household humidifier disinfectant and associated risk of lung injury (HDLI) in South Korea. | Science of The Total Environment (2017) | Park et al. | Estimate exposure of PHMG, CMIT/MIT |
